# Application of STEM tomography to investigate smooth ER morphology under stress conditions

**DOI:** 10.1111/jmi.70020

**Published:** 2025-08-12

**Authors:** V. Heinz, R. Rachel, C. Ziegler

**Affiliations:** ^1^ Department of Biophysics II/Structural Biology University of Regensburg Regensburg Germany; ^2^ Department of Molecular and Cellular Anatomy/Centre for Electron Microscopy University of Regensburg Regensburg Germany

**Keywords:** crystalloid‐ER, endoplasmic reticulum (ER) morphology, ER stress, ER whorls, scanning transmission electron microscopy (STEM), tomography

## Abstract

The endoplasmic reticulum (ER) is a highly dynamic organelle that undergoes significant morphological alterations in response to cellular stress. While conventional transmission electron microscopy (TEM) has provided valuable insights into these changes, such as the formation of crystalloid‐ER and ER whorls, obtaining comprehensive three‐dimensional (3D) information on these large structures within their cellular context has remained a challenge. To overcome these limitations, this study introduces an innovative application of dual‐axis scanning transmission electron microscopy (STEM) tomography to investigate ER morphology under stress conditions in human embryonic kidney (HEK) cells overexpressing the cation channel polycystin‐2 (PC‐2). Benefitting from high‐resolution, increased depth‐of‐focus, and reduced aberrations, STEM tomography enabled the detailed 3D reconstruction of large cellular subvolumes, providing unprecedented views of stress‐induced ER structures. Our findings reveal distinct ultrastructural details of both crystalloid‐ER and ER whorls. Crystalloid‐ER exhibited a tubular architecture with potential interconnectedness, while ER whorls displayed a lamellar organisation and distinct membrane curvature. We observed the co‐occurrence of these distinct smooth ER (sER) morphotypes within the same cell, yet they remained spatially separated, suggesting potential functional specialisation. Furthermore, we identified direct membrane contacts in mixed morphotypes, hinting at a shared origin or dynamic relationship between these structures. The study also elucidated the interactions of these organised smooth ER (OSER) structures with other organelles, such as mitochondria (MAM sites) and vesicles. In summary, the presented ultra‐structural insights have a significant impact on our understanding of stress‐related ER morphology changes. The ability to visualise the intricate 3D architecture and spatial relationships of these structures provides novel perspectives on the ER's adaptive responses to stress, including potential roles in lipid and protein biosynthesis and intracellular communication. These findings underscore the power of dual‐axis STEM tomography for elucidating complex organellar organisation and dynamics in their native cellular context.

## 1 INTRODUCTION

Eukaryotic cells constantly face a variety of stressors, both external and internal, and rely on sophisticated strategies to manage stress, such as the unfolded protein response (UPR).^1^, ^2^ The UPR reduces protein synthesis and enhances the cell's ability to fold proteins correctly when there is a buildup of unfolded or misfolded proteins in the endoplasmic reticulum (ER).^3^ During the UPR, the ER increases its luminal volume and membrane surface area.^4^ These additional membranes can form organised smooth ER (OSER) structures, which include lamellar and tubular shapes.^5^, ^6^, ^7^, ^8^ Tubular ER honeycomb‐like arrays, known as “crystalloid‐ER,” have been described in several studies.^6^, ^9^, ^10^, ^11^, ^12^ OSER structures can also appear in cells that are not under stress, as seen in highly active ER phases in differentiated secretory cells, such as plasma B cells or embryonic adrenal cells.^13^ While the exact mechanisms behind OSER formation are not fully understood, factors such as changes in membrane lipid and sterol composition, curvature‐forming proteins, and interactions with scaffolding proteins were suggested to play a role.^9^, ^14^, ^15^, ^16^

Detailed 3D information on OSER morphotypes is still scarce. Scanning Transmission Electron Microscopy (STEM) tomography has shown promise in offering higher resolution images of cellular structures.^17^, ^18^, ^19^, ^20^ Unlike TEM, which uses a broad, quasi‐parallel electron beam, in STEM, a finely focused, convergent beam scans the sample. As the beam interacts with the sample, it scatters electrons, which are captured by bright field (BF) and dark field (DF) STEM detectors. The information obtained for each scanned pixel are subsequently combined to a mosaic image.^17^ By operating in the ‘biological’ STEM mode, with a beam 2–3 nm in diameter, which is 10 times larger than in ‘standard’ STEM applications, thicker sections of up to 1 µm can be investigated even at high tilt angles (> ± 66°).^19^, ^21^, ^22^ In comparison to the TEM tomography approach, biological STEM benefits from a higher depth‐of‐focus,^19^ the possibility of performing dynamic focusing^23^ and the lack of chromatic aberration,^18^, ^19^, ^20^, ^24^, ^25^, ^26^, ^27^ as well as from a better Signal‐to‐noise‐ratio (SNR) and contrast.^28^ For biological STEM applications, a correction of the contrast transfer function is generally not considered necessary, and sections with a nominal thickness of up to 1 µm can be investigated even at high tilt angles (> ± 66°). The preparation of samples through high‐pressure freezing^29^, ^30^ and freeze substitution^31^, ^32^ ensures excellent preservation of cellular ultrastructure, offering significant advantages over chemical fixation.^19^, ^33^ Rapid freezing preserves cells, avoiding artefacts such as structural deformation that conventional methods may cause. Meanwhile, freeze substitution allows gentle chemical fixation that maintains cell integrity, preventing artefacts from washing out soluble cytoplasmic elements.^34^, ^35^ The method thus is ideally suited to investigate the organellar architecture of different OSER morphotypes in 3D.

In this study, we focus on the role and impact of OSER structures that form when the Transient Receptor Potential (TRP) channel Polycystin‐2 (PC‐2) is overexpressed in HEK293S cells^36^ mimicking UPR. Previous research using focused ion beam (FIB) scanning electron microscopy (SEM) revealed crystalloid‐ER formation and PC‐2 enrichment in these membranes.^36^ Here, we use STEM tomography to obtain 3D reconstructions and monitor the genesis and dynamic nature of crystalloid‐ER. This method provides better resolution in the Z‐dimension and reduces the missing wedge artefact common in single‐axis tomograms, allowing us to explore a larger cellular volume in detail.

## 2 MATERIAL AND METHODS

### 2.1 Cell culture and protein over‐expression

A stable HEK293S GnTI^−^ cell line^36^ was used for the expression of WT PC‐2. Cultivation of cells and expression of PC‐2 were performed as described in this publication. For subsequent HPF/AFS, plasma‐cleaned (Plasma Cleaner PDC‐3XG, Harrick Plasma Inc., Ithaca, USA) TC 60 cell culture dishes were supplemented with 1.4 mm sapphire discs (M. Wohlwend GmbH, Sennwald, CH) prior to seeding the cells. Cells were grown in DMEM/F‐12 medium (Sigma‐Aldrich®, St. Louis, USA), supplemented with 10% fetal bovine serum (FBS), 5 µg/mL blasticidin, 350 µg/mL G418, and Pen/Strep (55 µg/mL penicillin, 55 µg/mL streptomycin). At ∼40% confluence, protein expression was induced via addition of tetracycline to a final concentration of 3 µg/mL. The sapphire discs were transferred into sterile cell culture medium after 72 h of expression, at ∼80%–90% confluence. Noninduced cells were grown in parallel as a negative control.

### 2.2 High‐pressure freezing, freeze‐substitution and sectioning

Subsequently, the samples were prepared by high pressure‐freezing (HPF), freeze‐substitution, resin embedding and ultramicrotomy, in general following the procedures outlined by R. Rachel and co‐workers.^34^ Cryo‐fixation was performed via HPF at ∼2048 bar and –196°C in an EM PACT2 (Leica Microsystems GmbH, Wetzlar, DE), followed by automated freeze‐substitution in an AFS2 (Leica Microsystems GmbH, Wetzlar, DE). Intracellular water was initially substituted against 0.5% (v/v) OsO_4_, 0.25% (w/v) uranyl acetate (UAc), 5% (v/v) H_2_O in acetone, or 0.5% (v/v) glutardialdehyde (GA), 0.5% (w/v) UAc, 5% (v/v) H_2_O in acetone. OsO_4_‐stained samples were used for screening and tomography, samples stained with only UAc exclusively for screening. The latter were subjected to lead citrate (PbCi) staining on the sections later on, following common procedures. Notably, special care was taken to obtain the best ultrastructural preservation of the (intra‐) cellular membranes by adding H_2_O to the freeze substitution solution.^37^, ^38^ After polymerisation, HPF sample carriers and sapphire discs were removed from the resin blocks, and the samples were trimmed to a trapezoidal block‐face shape using fresh razor blades. Subsequently, ultrathin sections (50 nm) were prepared for sample screening and thick sections (800 nm) were prepared for STEM tomography on an EM UC7 (Leica Microsystems GmbH, Wetzlar, DE) ultramicrotome using a ‘histo’‐type diamond knife (Diatome Ltd, Nidau, CH). The sections were picked up on pioloform‐coated copper slot (1×2 mm) or P‐bar grids. For tomography, 15 nm protein A gold fiducials (University Medical Center Utrecht, Utrecht, NL) were successively applied to both sides of the sections at a dilution of 1:40, incubated for 4 min and excess gold particles were washed away with distilled H_2_O. Finally, a thin, conductive carbon film of 2–3 nm thickness was evaporated onto the grid and section surface via thermal evaporation in a Cressington 208 carbon (Tescan GmbH, Dortmund, DE).

### 2.3 Electron microscopy

Screening of grids for optimal ultrastructural sample preservation and for the presence of crystalloid ER on sections with a nominal thickness of 50 nm was performed by conventional TEM microscopy on a JEM 2100F (JEOL, Freising, DE) transmission electron microscope equipped with a field emission gun, operated at 200 kV with a CMOS camera (TemCam‐F416, 4k × 4k pixels, TVIPS GmbH, Gilching, DE) using the EM MENU (TVIPS – Tietz Video and Image Processing Systems GmbH, Gilching, DE) software package. Screening revealed good sample quality and excellent preservation of intracellular membranes. Sections with a nominal thickness of 800 nm were consequently analysed via dual‐axis STEM tomography, following the established procedures at the Department for Molecular and Cellular Anatomy at our university.^19^ Since plastic sections shrink substantially during data collection,^39^ the samples were preexposed to the electron beam for several minutes (‘beam showering’). Dual‐axis STEM tomography was performed on the same JEM 2100F (JEOL, Freising, DE) electron microscope equipped with both a bright field (BF) and a dark field (DF) STEM detector, using a Model 2040 dual‐axis tomography holder (Fischione Instruments Inc., Export, USA). The STEM beam was formed using special settings,^19^ with a 20 µm condenser aperture in place (diameter: 3–4 nm). Fine‐tuning was accomplished via a ronchigram generated on the pioloform support film. At a camera length of 20 cm, both BF and DF images were collected simultaneously; the signal was optimised for BF imaging. Tilt series were recorded automatically from +66° to –66° according to a modified Saxton tilt scheme^40^ presented in Table S2 using the EM‐Tools (TVIPS – Tietz Video and Image Processing Systems GmbH, Gilching, DE) software package. Eucentric height was adjusted once before data acquisition. After collection of the first tilt series completed, the sample was manually rotated in the holder by 90°. Subsequently, a second tilt series was acquired as described before from the same region of interest (ROI).

### 2.4 Tomogram reconstruction and visualisation

Finally, tomogram reconstruction was performed in IMOD, following the inherent processing pipeline^41^ and according to common procedures.^19^, ^42^ Preprocessing included binning and image stack generation. Fine tuning of the initial, coarse image alignment requires a fiducial model. Here, manual fiducial model generation and optimisation yielded superior results than automatically generated models. At least 15 fiducial markers were selected from each side of the section. Distortions were accounted for during later iterations of fine alignment. The volume was cropped, and gold fiducials were removed using the ‘Bead Eraser’ option. Tomogram reconstruction was performed by a (weighted) back projection including a radial filtering, with a SIRT‐like filter equivalent to 15 iterations, which in this case yielded the best results. Both tomograms collected from the same ROI were reconstructed following the procedure described above and subsequently merged using the ‘Tomogram Combination’ option. Alignment of the two tomograms usually worked best following the ‘automatic patch fitting’ method. Finally, the tomogram edges were trimmed. For tomogram visualisation, the program SeggeR^43^ in the UCSF Chimera^44^ framework could be identified as a threshold‐based approach, circumventing manual membrane tracing as commonly applied. The tomograms were imported into UCSF Chimera, scaled and binned appropriately, and the histogram display threshold was adjusted to optimally visualise the organellar membranes. Subsequently, SeggeR was used for automated, threshold‐based segmentation of the volume. Two or three smoothing steps were performed, very small regions (background noise < 5 to 30 voxels) were removed, and the segmentation results were refined manually. The ‘Ungroup’ option was used to improve the quality of inaccurately segmented regions, and regions belonging to the same organelle were subsequently grouped together to obtain one separate volume per organelle.

## 3 RESULTS

### 3.1 Detailed 2D characterisation of ER whorls and putative crystalloid‐ER structures by TEM

The morphology of HEK293S cells after 72 h of overexpression of full‐length WT PC‐2 was firstly investigated using conventional transmission electron microscopy (TEM) (Figure S1). As a control experiment, noninduced cells were analysed and exhibited typical morphology without any obvious abnormalities, indicating that the cellular ultrastructure was preserved during sample preparation. Under stress conditions, the nuclei showed areas of highly electron‐dense, condensed chromatin. Lamellar endoplasmic reticulum (ER) structures composed of stacked membrane sheets were identified close to the nuclei and classified as karmellae due to their characteristic morphology and localisation (arrow in Figure S1a).^8^, ^45^ We observed densely packed areas with polymorphic vesicles (Figure S1b), which varied in size and were enclosed by well resolved membranes. Circular arrays of stacked lamellar membranes with regular spacing appeared in the cytoplasm (Figure S1c), mostly located near the nucleus, with mitochondria frequently nearby. Based on their characteristic morphology and subcellular localisation, these membrane arrays were identified as ER whorls, another lamellar OSER morphotype.^5^ Finally, patches of regular, putatively tubular membrane arrays could be observed^12^ (Figure S1d). However, in 800 nm sections, tubular structures were difficult to depict, and it was not possible to discriminate between tubular, sinusoidal or hexagonal crystalloid ER. None of these OSER structures were observed in the control experiment without PC‐2 overexpression. Different types of OSER, mostly whorls and crystalloid‐ER, could be detected in only approximately 10% of cells.

Thinner sections of the sample (50 nm) were investigated at higher magnifications. A collection of various whorls is depicted in Figure S2. These whorls are composed of stacked, lamellar membrane sheets arranged in either open (Figure S2a) or closed (Figure S2b–d) circular formations reaching diameters of up to 2 µm. It is important to note that both forms may potentially represent different sections of the same organelle, as indicated in Figure S2c. The presence of open‐circle whorls has been previously described as loop‐like whorls.^6^ The edges of whorl sheets in open circles are created by membranes wrapping back by 180°, resulting in extreme curvature (Figure S2a). The stacked membranes display consistent spacing, more prominent in closed‐circle whorls (Figure S2d). Multiple whorls can be observed within a single cell, with vesicles often found enclosed by or in the vicinity of the circular membrane stacks (Figure S2a and c). The direct fusion or fission of these vesicles with the ER membranes remains uncertain. Additionally, mitochondria are frequently located near the whorls, aligning with previous research on the clustering of mitochondria around OSER structures.^6^ While whorls are commonly found in close proximity to the nucleus, direct connections between whorls and the nuclear envelope or whorls and karmellae were not observed. In lead‐citrate contrasted sections of *en bloc* uranyl acetate‐stained samples, electron‐dense spots resembling ribosomes can be seen occasionally lining the peripheral membrane sheets (arrow in Figure S2d and e). These spots are more prevalent in less organised whorl‐like structures and exhibit the characteristic appearance of ribosome‐decorated rough ER (rER) membranes. This discovery suggests an organisation of OSER structures into distinct functional subdomains, in line with previous reports.^46^ Figure S3 illustrates two examples of putative crystalloid ER structures. The observed tubular patches reached diameters of up to 2.5 µm and were often located near the nucleus and close to mitochondria, like the observed whorls. The membranes displayed a regular pattern; however, the precise symmetric arrangement could not be determined from the section planes provided. Unlike the lamellar whorls, the spaces between the tubular arrays contained less cytoplasmic material. Like whorl structures, multiple tubular OSER organelles can be present in a single cell. A 3D analysis is needed to determine whether they are separate or interconnected.

### 3.2 3D reconstructions reveal vesicle entrapment in ER whorls and close association with mitochondria

**FIGURE 1:**
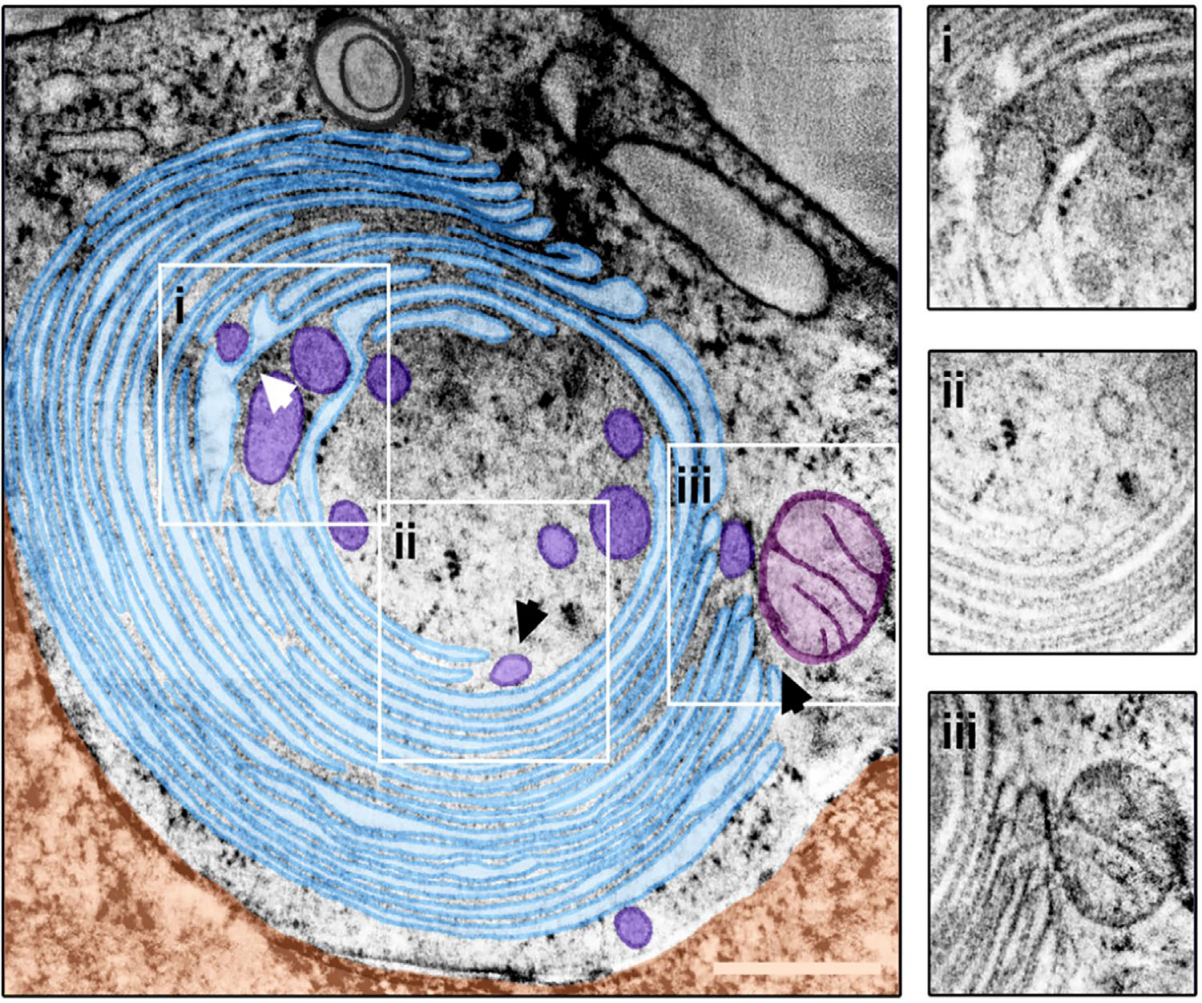
STEM tomography of ER whorls. Screening of the reconstructed STEM tomogram of a whorl (light blue) resulted in three ROIs, marked by boxes i–iii. The selected areas putatively show the integration of vesicles (dark blue) into the organelle (I), fusion or budding of a terminal vesicle (II), and interactions with a mitochondrion (purple) (III). An interaction between the nucleus (orange) and the whorl was not evident from the tomogram. Arrowheads indicate the viewing direction in the 3D close‐ups depicted in Figure 2. Scale bar = 500 nm.

**FIGURE 2:**
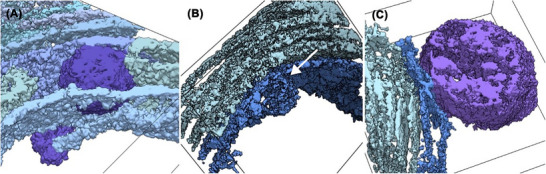
ER whorl morphology in 3D. Subvolumes of a tomogram were segmented and rendered in 3D to visualise details of the ER whorl morphology. (A) A vesicle (dark blue) embedded into a highly ordered, regular array of stacked whorl membranes (light blue). (B) Vesicle fusion or fission to/from a whorl membrane. (C) Direct interaction between whorl membrane (blue) and mitochondrion (violet). Black outlines indicate the subtomogram boundaries.

To perform a 3D analysis, dual‐axis STEM tomography was conducted on 800 nm sections. Various types of OSER (lamellar, tubular, and mixed morphologies) were examined. In every case, the structures were found to be located near the nucleus and associated with mitochondria. A detailed examination of the tomograms revealed additional structural details. Three regions of interest (ROIs) were identified in a reconstructed whorl tomogram (Figure 1) highlighting interactions with multiple vesicles and a mitochondrion. However, no direct connection to the nucleus was observed in the imaged subvolume. Segmentation of the subvolumes (Figure 1I–III) provided further insights into whorl interactions. Ordered stacked membrane sheets which make up a major part of the whorl, envelop multiple vesicles (Figure 2A).

ER whorl and vesicle membranes are in direct contact and fusion and fission of a vesicle with or from the terminal point of a whorl membrane were observed (Figure 2B). Several dozen vesicles were identified in contact to the whorl membranes. A mitochondrion is located at the whorl periphery in direct interaction with it, although no fusion processes were observed between the organelles (Figure 2C). This contact site shows the typical features of a mitochondria‐associated membrane (MAM) site, as described by Baumann & Walz.^47^

**FIGURE 3:**
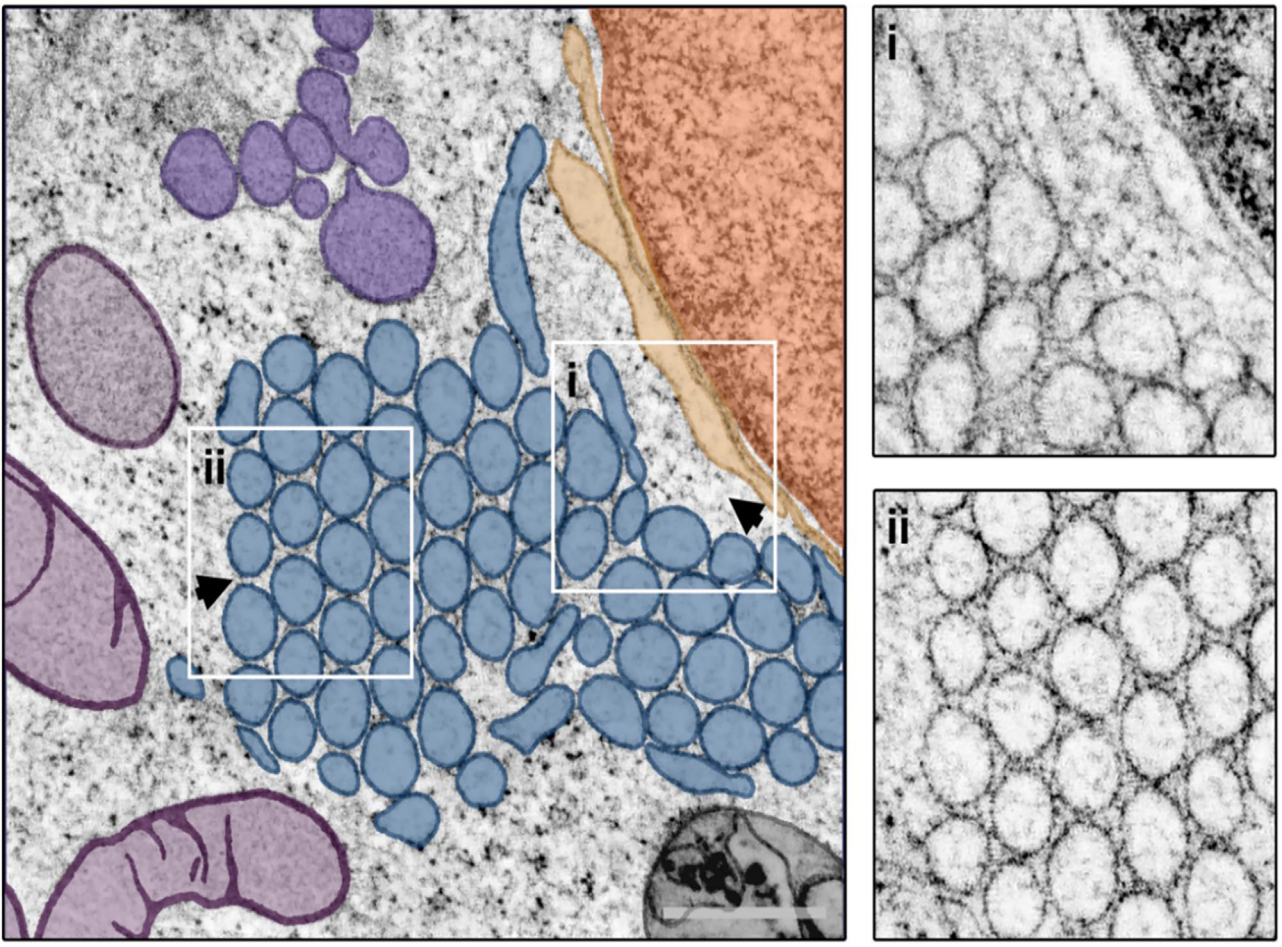
Crystalloid‐ER morphology. The crystalloid‐ER (blue), formed by a regular array of tubular ER membranes, is in close vicinity to nucleus (dark orange) and nuclear envelope (orange) and surrounded by multiple mitochondria (violet) and vesicular structures (violet blue, grey). Putative interactions between crystalloid‐ER and the nuclear envelope (I) as well as the tubular nature of the crystalloid‐ER itself (II) were analysed further via segmentation and 3D visualisation of the tomogram. Arrowheads indicate the viewing direction in Figure 4. Scale bar = 500 nm.

A comprehensive 3D analysis was conducted to uncover structural details of the tubular ER morphotype. The intracellular positioning of the crystalloid‐ER patch, as shown and analysed in Figure 3 closely resembles the placement of the ER whorl previously described. This patch, located near the nucleus, is surrounded by several mitochondria and vesicular structures. The reconstructed tomogram revealed a vesicle situated between the crystalloid‐ER and the nucleus in the area depicted in Figure 3I, which is not apparent in the central tomogram slice. Consequently, segmentation and 3D rendering were employed to provide a clearer spatial understanding of the crystalloid‐ER itself (Figure 3ii) and its interactions within the cell.

**FIGURE 4:**
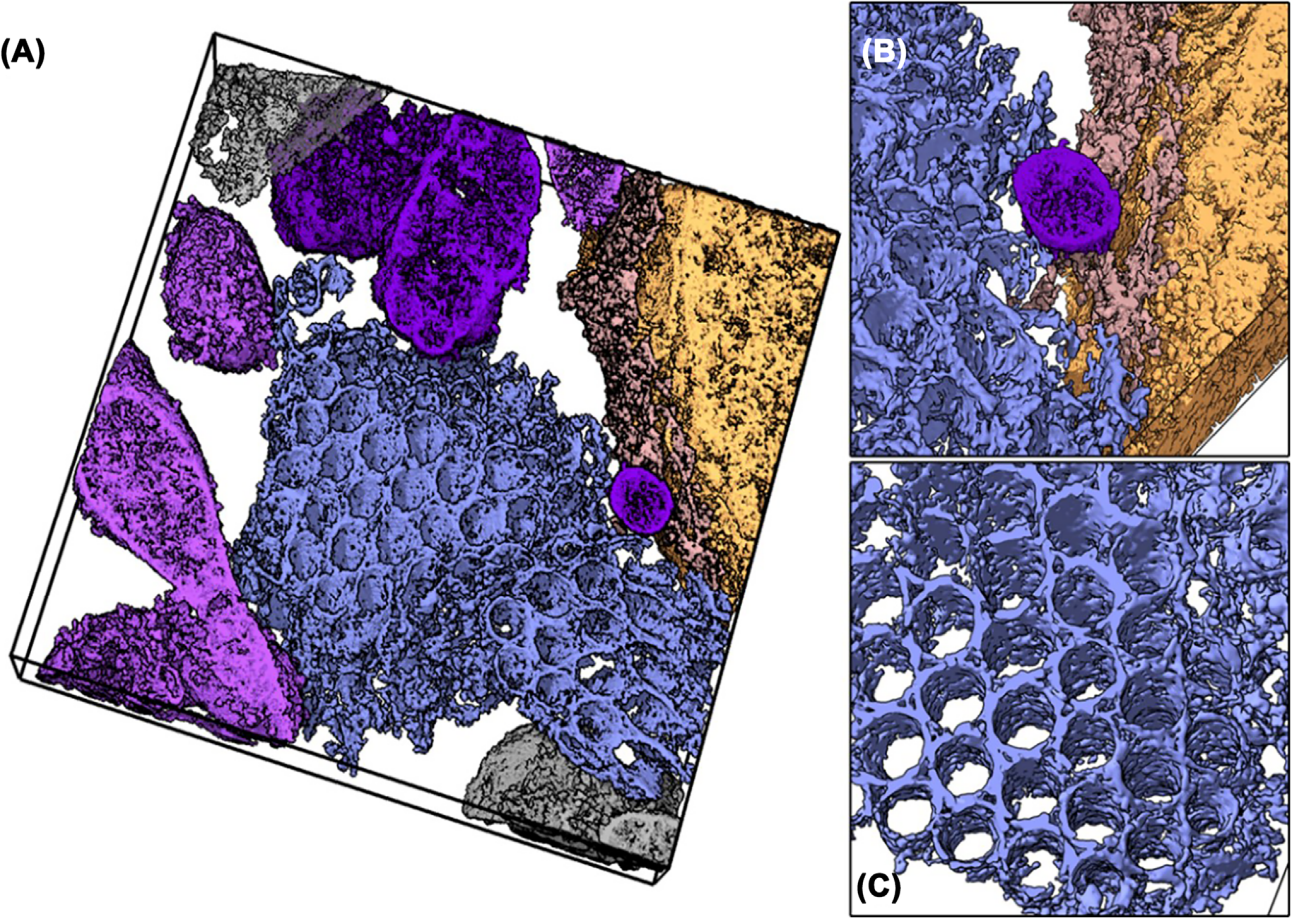
Crystalloid‐ER morphology in 3D. (A) Segmented and 3D‐rendered visualisation of the crystalloid‐ER (blue) in its cellular context, surrounded by multiple mitochondria (violet). (B) Detail of a vesicle (dark blue) mediating contact between the crystalloid‐ER (blue) and the nuclear envelope (brown). (C) Cut‐open detail of the crystalloid‐ER, highlighting the regular nature of the tubular ER membrane array.

The crystalloid‐ER does not make direct contact with the surrounding mitochondria but closely approaches the nuclear envelope (Figure 4A). A single vesicle is present in the contact area, interacting with both the nuclear envelope and the crystalloid‐ER (Figure 4B). However, there is no evidence of fusion or fission involving this vesicle and either organelle. Unlike the previously described ER whorl, there are no vesicles embedded within the crystalloid‐ER patch.

The tubular structure and orderly arrangement of the crystalloid‐ER membranes are most clearly observed in the cut‐open view (Figure 4C). According to the reconstructed tomogram (Figure 3) and 3D visualisation, each crystalloid‐ER tube is enclosed by its membrane. These tubes are organised in rows with a consistent offset of half a tube's diameter, maximising space efficiency. In sections, the interior of the tubes, corresponding to the ER lumen, appears electron lucent. Consistently, the reconstructed tomogram shows minimal density blobs. In contrast, the spaces between the tubes are often filled with electron‐dense cytoplasmic material and are always bounded by the membranes of three neighbouring tubes.

**FIGURE 5:**
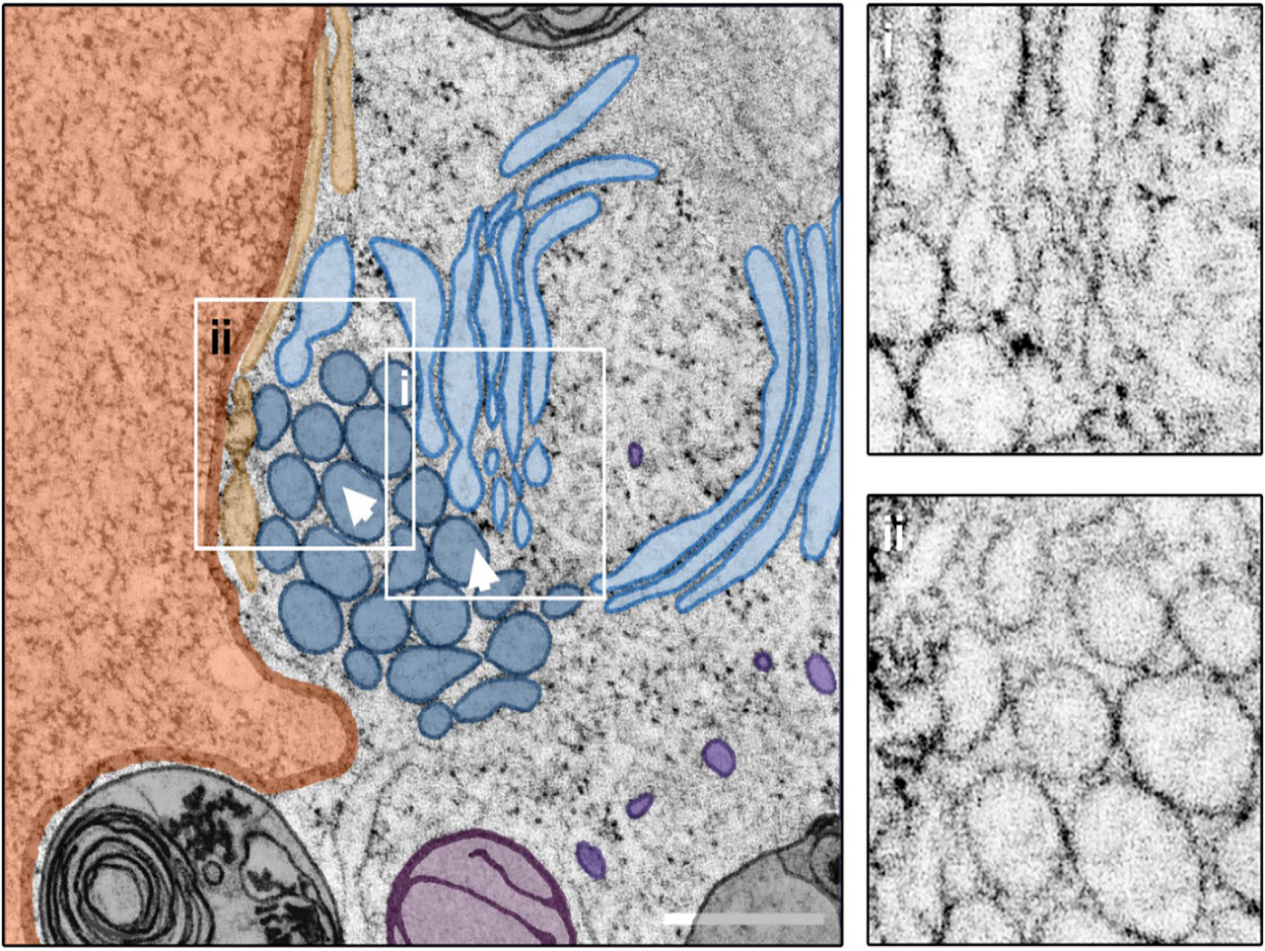
Mixed crystalloid‐ER and whorl morphology. Central slice through a STEM tomogram showing a patch of crystalloid‐ER (dark blue) located in close proximity to a putative ER whorl (light blue) in its intracellular environment. Closer inspection of the reconstructed tomogram reveals a direct connection between the two ER morphotypes (I), as well as the presence of multiple vesicles (not present in the central tomogram section), located near the nucleus (orange) and contacting the crystalloid ER (II). Arrowheads indicate the viewing direction in Figure 6. Scale bar = 500 nm.

In a single cell, both whorls and crystalloid‐ER are present simultaneously (Figure 5). Interestingly, within this mixed morphotype, only the crystalloid‐ER directly interacts with the nuclear envelope, whereas the whorl is positioned farther from the nucleus. Notably, the crystalloid‐ER and whorl are positioned adjacent to each other in the mixed morphotype observed here (Figure 5I). Additionally, numerous vesicles and vesicular structures are located near the ER, with some contacting the crystalloid‐ER (Figure 5II).

**FIGURE 6:**
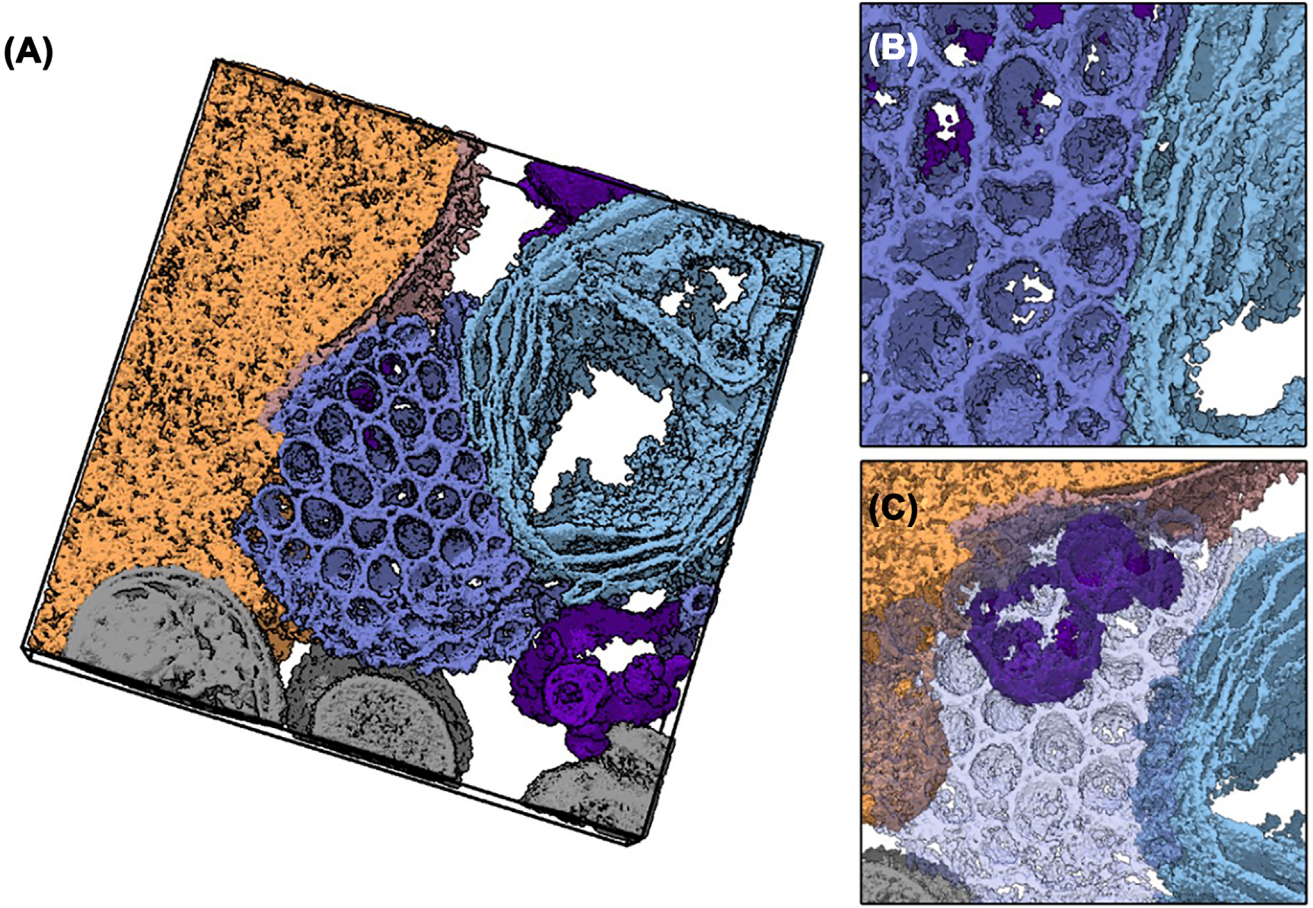
Mixed ER morphology in 3D. (A) Segmentation and 3D rendering of a mixed ER morphotype consisting of a patch of crystalloid‐ER (dark blue) and an adjacent ER whorl (light blue). Both are located in proximity to the nucleus (orange) and nuclear envelope (brown) and surrounded by multiple vesicles (violet). (B) The direct contact between the two ER morphotypes becomes evident from the 3D visualisation. (C) The tight association of crystalloid‐ER (transparent), nucleus and vesicles.

Further insights into the complex organisation of the ER membranes in the mixed morphotype are revealed through segmentation and 3D visualisation (Figure 6A). The tubular crystalloid‐ER membranes and the lamellar whorl membrane sheets can be clearly distinguished and traced throughout the tomogram's volume. Remarkably, the crystalloid‐ER and whorl exhibit a pronounced interaction site with noticeable direct membrane contacts (Figure 6B) highlighting the shared origin of these morphologically distinct organelles. While some crystalloid‐ER tubes extend through the entire tomogram volume, others only reach about halfway and are partially capped by slightly domed membranes at their endpoints, suggesting a completely enclosed crystalloid‐ER lumen. This observation is further supported by the electron‐lucent appearance of the crystalloid‐ER tubes' interior in sections (Figure S3). As previously described for individual organelles, in the mixed morphotype, ER patches are surrounded by and closely interact with multiple vesicles and vesicular structures. In the subvolume of the cell examined here, a prominent vesicle cluster is identified near both the crystalloid‐ER and the nucleus, potentially serving as an interaction platform between these two organelles (Figure 6C).

### 3.3 Comparative 3D STEM morphometry of crystalloid‐ER tubes and ER whorl lamellae

**FIGURE 7:**
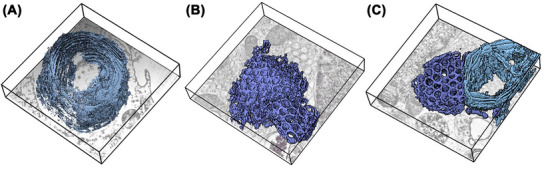
Crystalloid‐ER and whorl dimensions in HEK cells upon over‐expression of PC‐2 ER whorl (light blue) and crystalloid‐ER (dark blue) volumes are displayed for the three STEM tomograms (A–C) in 3D. The tomogram dimensions are indicated by black boxes, the bottom tomogram slices are depicted for a better spatial orientation. The depictions consistently highlight the overwhelming abundance of the ER morphotypes in the respective cell subvolumes.

ER whorls and crystalloid‐ER patches are noticeably larger compared to the ER of noninduced HEK293S cells (Figure 7). These ER patches extend throughout the entire height (Z‐axis) of the tomogram, exceeding the section thickness of 800 nm. Conversely, the maximum planar dimensions of these organelles range from approximately 1.4 to 2.2 µm for each morphotype. As the entire dimensions of the organelles cannot be captured in a single section, their exact volumes cannot be determined through STEM tomography. Additionally, it should be noted that multiple whorls or crystalloid‐ER patches can exist within a single cell, thereby increasing the total organelle volume. While the ER volume and ER membrane surface area increase significantly during the overexpression of WT PC‐2 in HEK293S cells, the overall cellular volume remains constant. ER whorls and crystalloid‐ER consistently remain separate and do not intersect or alternate; no intermediate forms were observed in the imaged subvolumes. The distinct separation of sheets and tubes, along with the consistency of their disparate shapes, suggests an underlying shaping mechanism. Detailed examination of the tomograms shows that the crystalloid‐ER tubes have a nearly perfect circular cross‐section in the X‐Y plane with a uniform diameter of about 160 nm (average of ten tubes) (Figure S4a). Each individual tube is encircled by six additional tubes, creating an approximately triangular cross‐section in the space between them. Hypothetical linkages among these interspaces produce an apparent hexagonal symmetry in the arrangement of the crystalloid‐ER membranes (Figure S4b). This results in a uniform array of tubes, dictated by the moderate curvature of the membrane in the *X*–*Y* plane. In the *Z*‐direction, curvature is confined to the terminal caps of the crystalloid‐ER tubes.

In stark contrast to the crystalloid‐ER, whorls consist of stacked membrane sheets arranged in a regular, lamellar configuration (Figure S4c). At their terminal points, these membrane sheets loop back in a 180° turn, enclosing a very narrow ER lumen. This structure results in two distinct types of membrane curvature within the whorls (Figure S4d). While most of the whorl membranes display only slight curvature, the terminal turns exhibit extreme curvature, which is oriented oppositely to that of the rest of the membrane. According to prior classifications, these regions exhibit high positive membrane curvature relative to the cytoplasm.^15^ Unlike the crystalloid‐ER, the low curvature in the whorl membrane stack is not confined to the X‐Y plane but also extends into the Z dimension, likely giving the entire organelle an almost globular shape in 3D.

It is important to note, however, that not all whorls examined in this study present the same degree of order (see also Figure S2). The ER lumen is often described as a continuous space, offering the ER an environment specifically suited for its biochemical needs. Vesicles have been observed only near the crystalloid‐ER but are not integrated into the organelle itself. Therefore, direct connections between adjacent crystalloid‐ER tube membranes appear to be the most straightforward way to maintain a continuous ER lumen. Indeed, a potential connection site was identified in a tomogram slice (Figure S5a). By applying a threshold‐based isosurface representation to the relevant tomogram subvolume, an unbiased depiction of the crystalloid‐ER membranes was achieved (Figure S5b). A tilted view (Figure S5c) reveals the narrow connection between the two tubes, approximately 20 nm high, suggesting that direct connections between crystalloid‐ER tubes result in a continuous ER lumen. Although such a connection was not seen in a whorl, it should be noted that whorl membranes are often found in close proximity, which likely facilitates some sort of contact. It seems plausible that the whorl also maintains a continuous ER lumen. Additionally, multiple vesicles were identified within the whorls, indicating further potential for cargo exchange between the whorl lamellae.

## 4 DISCUSSION

### 4.1 Dual‐axis STEM tomography to investigate organellar architecture in 3D

The application of dual‐axis STEM tomography at 200 kV has been previously established as an effective method for visualising cellular structures in 3D at high resolution.^19^, ^22^ In our study, we further demonstrate its capability to resolve the intricate membrane architecture of organized smooth endoplasmic reticulum (OSER) structures, surpassing the resolution achieved in our earlier FIB‐SEM analysis of the same samples,^36^ as well as recent FIB‐SEM analyses and 3D reconstructions of ER whorls in mammalian cells.^48^ Historically, freeze‐fracture studies suggested a tubular nature of the crystalloid‐ER in UT‐1 cells, providing a broad 3D impression without the ability to discern individual membranes.^9^ In contrast, our current investigation, involving reconstructed cellular volumes of 800 nm, clearly identifies membrane‐enclosed tubes within the crystalloid‐ER and lamellar membrane sheets within ER whorls. This is achieved by tracing individual ER membranes with precision (Figures 1, 2 and 5). The dual‐axis STEM tomography of plastic‐embedded samples effectively bridges the gap between cryo‐TEM tomography of isolated organelles and FIB‐SEM analysis of organelles in the cellular context, all at nanometer resolution. This method provides a middle ground between these techniques and super‐resolution light microscopy. In summary, the method has yielded significant insights into the complex intracellular interaction networks of OSER structures, allowing us to visualise their membrane architecture with extraordinary detail.

### 4.2 Whorls as integral parts of the ER network

ER whorls and crystalloid‐ER appear in similar size and quantity when PC‐2 is overexpressed in HEK293S cells. Additionally, a mixed crystalloid‐ER and whorl morphology has been observed (Figures 5 and 6). ER whorls are directly associated to mitochondria (Figure 2C), identifying the whorl membrane interface as a MAM site – an ER domain closely involved in lipid biosynthesis.^47^ This suggests that ER whorls may play a role in lipid biosynthesis to satisfy the increased demand for lipids required to assemble the substantial amount of bulk ER found in both crystalloid‐ER and whorls (Figure 7). Ribosomes were identified at peripheral ER whorl membrane sheets (Figure S2). These findings indicate that ER whorls may function as sites of active protein and lipid biosynthesis, with the lamellar whorl membrane sheets resulting from ongoing ER membrane expansion. It has been reported that a global increase in the yeast ER membrane is stimulated by UPR signalling and achieved through the expansion of lamellar membrane sheets to mitigate ER stress.^4^ This raises the question of whether the pronounced OSER morphotypes seen with PC‐2 overexpression represent an acute, dangerous stress condition for the cell or a routine cellular response to alleviate and manage such stress. Recent studies have shown that PC‐2 upregulation occurs under pathological conditions across various tissue types, including the kidney, liver, brain and heart, in both human and animal models. Conversely, cells with PC‐2 knockdown or knockout showed increased vulnerability to stress‐induced cell death.^49^ Therefore, the pronounced OSER morphotypes may serve as a universal stress response to increase cell survival.

### 4.3 Protein interactions driving crystalloid‐ER assembly

Crystalloid‐ER formation has been widely observed both in vivo and under conditions of protein inhibition or over‐expression across multiple systems.^47^ Our tomographic analysis has now, for the first time, revealed the tubular nature of crystalloid‐ER with a resolution that allows us to trace ER membranes and distinguish individual crystalloid‐ER tubes. These tubes are shown to be interconnected (Figures 2, 4, and S5), supporting the concept of a continuous crystalloid‐ER lumen. There is currently no ultrastructural evidence indicating active protein biosynthesis within the crystalloid‐ER; instead, it appears that mature PC‐2 accumulates in crystalloid‐ER patches.^36^

Crystalloid‐ER proliferation from lamellar ER membrane sheets has been observed in UT‐1 cells when expressing increased levels of 3‐hydroxy‐3‐methylglutaryl coenzyme A (HMG‐CoA) reductase, the enzyme that serves as the rate‐limiting step in cholesterol biosynthesis.^50^ Similarly, in COS cells, over‐expression of mouse microsomal aldehyde dehydrogenase (msALDH) led to crystalloid formation. Notably, truncated versions of the msALDH protein did not result in crystalloid formation, much like PC‐2 C‐terminal truncations.^12^, ^51^ It appears that crystalloid‐ER formation is mediated through homotypic soluble domain interactions.^6^, ^47^ Supporting this hypothesis, membrane‐protein‐induced ER membrane curvature has been demonstrated both in vitro and in vivo.^14^

### 4.4 Functional implications of crystalloid‐ER and whorls in the cell

Both crystalloid‐ER and whorls occupy a considerable portion of the cells' volume, raising questions about the functional status of the expanded ER. At the time of investigation, the cultivated cells exhibited no significant differences in overall fitness compared to the control group, as assessed by indicators such as the presence of dead cells and detachment of adherent cells from the culture dish. The ER is consistently described as the largest cellular membrane system,^52^ with ample evidence supporting the concept of an entirely continuous ER lumen.^6^, ^47^ Our ultrastructural STEM analyses consistently revealed the presence of an intact interaction network for all ER morphotypes, including the nuclear envelope, vesicles, and mitochondria, facilitating multiple possibilities for intracellular communication and interaction. This aligns with observations of a distinct co‐localisation of ER subdomains with their intracellular interaction partners.^53^ These observations suggest that both ER whorls and crystalloid‐ER function as active organelles. However, crystalloid‐ER and whorls are two very distinct morphotypes. Despite the existence of a mixed morphotype, no intermediate forms, intersections between the two types, or altered tube‐lamella arrays were detected in the sample. Thus, crystalloid‐ER tubes and lamellar whorl membrane sheets are separated from each other. These findings suggest a specifically, orchestrated formation process, implying that these morphotypes represent subdomains performing different tasks within the ER. Furthermore, the ER itself is divided into the nuclear envelope and peripheral ER, consisting of rER and sER. In this study, ribosome‐like structures were identified on some peripheral membrane sheets in less well‐ordered whorls (Figure S2) but were not detected in crystalloid‐ER patches. Moreover, a contact to a mitochondrion and the formation of the MAM site could be observed exclusively for whorls (Figure 3C). Taken together, these findings point towards distinct and different functions of crystalloid‐ER and whorls.

### 4.5 Functional roles of vesicle and mitochondria interactions with stress‐induced ER structures

The close association of vesicles with both ER whorls and crystalloid‐ER suggests important roles in cellular processes. In the case of ER whorls, the observation of multiple vesicles in direct contact with the whorl membranes and the potential for vesicle fusion or budding indicate that ER whorls are actively involved in vesicle trafficking. Furthermore, the presence of vesicles enveloped by the whorl membranes hints at a potential role in intra‐whorl cargo exchange or storage. Given that ER stress often involves an increased load of unfolded proteins, the interaction with vesicles could reflect the ER's attempts to manage this load through enhanced protein folding, degradation pathways, or transport of stress‐related molecules.

The observation of a prominent vesicle cluster located near both the crystalloid‐ER and the nucleus in mixed morphotype cells suggests a role for vesicles in mediating communication or transport between these two organelles, presumably involved in signalling related to the stress response or the regulation of crystalloid‐ER formation and function.

A consistent finding was the close association of both ER whorls and crystalloid‐ER with mitochondria. Notably, the direct contact between ER whorls and mitochondria, exhibiting features characteristic of mitochondria‐associated membranes (MAMs), suggests that these structures may be particularly important for local lipid synthesis, potentially to meet the increased demand for membrane lipids required for the formation and maintenance of the extensive ER networks seen in both whorls and crystalloid‐ER under stress conditions. While our study did not directly investigate calcium signalling at these sites, the established role of MAMs in this process^54^ suggests a potential involvement of ER whorls in regulating mitochondrial function and cellular calcium homeostasis during stress. The lack of observed fusion events between whorls and mitochondria indicates that the interaction is presumably focused on exchange of molecules and signals rather than structural integration.

In conclusion, the intricate interactions of vesicles and mitochondria with stress‐induced ER structures like crystalloid‐ER and ER whorls highlight the dynamic and interconnected nature of cellular organelles in response to stress. The spatial organisation and interactions observed underscore the ER's remarkable plasticity and its capacity to form specialised subdomains that cooperate with other organelles to maintain cellular homeostasis under challenging conditions.

## 5 CONCLUSION

In conclusion, our study has successfully employed dual‐axis STEM tomography to investigate the intricate three‐dimensional morphology of endoplasmic reticulum (ER) whorls and crystalloid‐ER structures formed in HEK cells under stress conditions induced by PC‐2 overexpression. Our work has provided unprecedented 3‐dimensional ultrastructural insights into these organised smooth ER (OSER) morphotypes, revealing key features such as the tubular nature and interconnectedness of crystalloid‐ER, the lamellar organisation and membrane curvature of ER whorls, and the spatial relationship and interactions between these structures, as well as with other organelles like mitochondria and vesicles. Notably, the ability to visualise the co‐occurrence yet spatial separation of these distinct ER morphologies within the same cell, along with the direct membrane contacts observed in mixed morphotypes, offers innovative insights into the dynamic organisation and potential functional specialisation of the ER under stress. These detailed morphological findings and the resulting insights into the ER's adaptive responses were made possible by the technical advantages of dual‐axis STEM tomography. Specifically, the technique's capacity for high‐resolution 3D imaging of relatively thick cellular sections, coupled with the benefits of increased depth‐of‐focus, minimal spherical and chromatic aberration, and improved signal‐to‐noise ratio compared to conventional TEM tomography, allowed for the comprehensive analysis of larger cellular subvolumes. Therefore, our study underscores the power of STEM tomography as a vital tool for elucidating complex organellar architecture within its native cellular context.

## AUTHOR CONTRIBUTIONS

VH and CZ: Conceptualisation. RR: Methodology. VH and RR: Investigation. VH: Formal analysis, visualisation, writing – original draft. VH, CZ, and RR: Writing – review and editing. CZ: Project administration.

## CONFLICT OF INTEREST STATEMENT

The authors declare no conflict of interest.

## Supporting information



Supporting Information

## References

[1] Harding, H. P. , & Ron, D. (2002). Endoplasmic reticulum stress and the development of diabetes. Diabetes, 51(3), S455–S461. 12475790 10.2337/diabetes.51.2007.s455

[2] Schröder, M. , & Kaufman, R. J. (2005). The mammalian unfolded protein response. Annual Review of Biochemistry, 74, 739–789. 10.1146/annurev.biochem.73.011303.074134 15952902

[3] Walter, P. , & Ron, D. (2011). The unfolded protein response: from stress pathway to homeostatic regulation. Science, 334, 1081–1086. 22116877 10.1126/science.1209038

[4] Schuck, S. , Prinz, W. A. , Thorn, K. S. , Voss, C. , & Walter, P. (2009). Membrane expansion alleviates endoplasmic reticulum stress independently of the unfolded protein response. Journal of Cell Biology, 187(4), 525–536. 19948500 10.1083/jcb.200907074 PMC2779237

[5] Koning, A. J. , Roberts, C. J. , & Wright, R. L. (1996). Different subcellular localization of *Saccharomyces cerevisiae* HMG‐CoA reductase isozymes at elevated levels corresponds to distinct endoplasmic reticulum membrane proliferations. Molecular Biology of the Cell, 7, 769–789. 8744950 10.1091/mbc.7.5.769 PMC275929

[6] Snapp, E. L. , Hegde, R. S. , Francolini, M. , Lombardo, F. , Colombo, S. , Pedrazzini, E. , Borgese, N. , & Lippincott‐Schwartz, J. (2003). Formation of stacked ER cisternae by low affinity protein interactions. Journal of Cell Biology, 163(2), 257–269. 14581454 10.1083/jcb.200306020 PMC2173526

[7] Voeltz, G. K. , Rolls, M. M. , & Rapoport, T. A. (2002). Structural organization of the endoplasmic reticulum. Embo Reports, 3(10), 944–950. 12370207 10.1093/embo-reports/kvf202 PMC1307613

[8] Wright, R. , Basson, M. , D'Ari, L. , & Rine, J. (1988). Increased amounts of HMG‐coa reductase induce “Karmellae”: A proliferation of stacked membrane pairs surrounding the yeast nucleus. Journal of Cell Biology, 107, 101–114. 3292536 10.1083/jcb.107.1.101 PMC2115167

[9] Anderson, R. G. W. , Orci, L. , Brown, M. S. , Garcia‐Segura, L. M. , & Goldstein, J. L. (1983). Ultrastructural analysis of crystalloid endoplasmic reticulum in UT‐1 cells and its disappearance in response to cholesterol. Journal of Cell Science, 63, 1–20. 6685129 10.1242/jcs.63.1.1

[10] Chin, D. J. , Luskey, K. L. , Anderson, R. G. , Faust, J. R. , Goldstein, J. L. , & Brown, M. S. (1982). Appearance of crystalloid endoplasmic reticulum in compactinresistant Chinese hamster cells with a 500‐fold increase in 3‐hydroxy‐3‐methylglutaryl‐coenzyme A reductase. PNAS, 79, 1185–1189. 6951166 10.1073/pnas.79.4.1185 PMC345926

[11] Takei, K. , Mignery, G. , Mugnaini, E. , Südhof, T. , & De Camilli, P. (1994). lnositol 1,4,5‐trisphosphate receptor causes formation of ER cisternal stacks in transfected fibroblasts and in cerebellar Purkinje cells. Neuron, 12, 327–342. 8110462 10.1016/0896-6273(94)90275-5

[12] Yamamoto, A. , Masaki, R. , & Tashiro, Y. (1996). Formation of crystalloid endoplasmic reticulum in COS cells upon overexpression of microsomal aldehyde dehydrogenase by cDNA transfection. Journal of Cell Science, 109, 1727–1738. 8832395 10.1242/jcs.109.7.1727

[13] Federovitch, C. M. , Ron, D. , & Hampton, R. Y. (2005). The dynamic ER: Experimental approaches and current questions. Current Opinion in Cell Biology, 17, 409–414. 15975777 10.1016/j.ceb.2005.06.010

[14] Hu, J. , Shibata, Y. , Voss, C. , Shemesh, T. , Li, Z. , Coughlin, M. , Kozlov, M. M. , Rapoport, T. A. , & Prinz, W. A. (2008). Membrane proteins of the endoplasmic reticulum induce high‐curvature tubules. Science, 319(5867), 1247–1250. 18309084 10.1126/science.1153634

[15] McMahon, H. T. , & Gallop, J. L. (2005). Membrane curvature and mechanisms of dynamic cell membrane remodelling. Nature, 438, 590–596. 16319878 10.1038/nature04396

[16] Stowell, M. H. B. , Marks, B. , Wigge, P. , & McMahon, H. T. (1999). Nucleotide‐dependent conformational changes in dynamin: Evidence for a mechanochemical molecular spring. Nature Cell Biology, 1, 27–32. 10559860 10.1038/8997

[17] Elbaum, M. (2018). Quantitative cryo‐scanning transmission electron microscopy of biological materials. Advanced Materials, 30(1706681), 1–6. 10.1002/adma.201706681 29748979

[18] Hohmann‐Marriott, M. F. , Sousa, A. A. , Azari, A. A. , Glushakova, S. , Zhang, G. , Zimmerberg, J. , & Leapman, R. D. (2009). Nanoscale 3D cellular imaging by axial scanning transmission electron tomography. Nature Methods, 6, 729–731. 19718033 10.1038/nmeth.1367 PMC2755602

[19] Rachel, R. , Walther, P. , & Maaßen, C. (2020). Dual‐axis STEM tomography at 200 kV: Setup, performance, limitations. Journal of Structural Biology, 211(3), 107551. 32589927 10.1016/j.jsb.2020.107551

[20] Wolf, S. G. , Shimoni, E. , & Elbaum, M. , et al. (2018). STEM tomography in biology. In Hanssen, E. (Ed.), Cellular imaging. Biological and medical physics, biomedical engineering (pp. 33–60). Springer.

[21] Biskupek, J. , Leschner, J. , Walther, P. , & Kaiser, U. (2010). Optimization of STEM tomography acquisition – A comparison of convergent beam and parallel beam STEM tomography. Ultramicroscopy, 110, 1231–1237. 20570046 10.1016/j.ultramic.2010.05.008

[22] Walther, P. , Bauer, A. , Wenske, N. , Catanese, A. , Garrido, D. , & Schneider, M. (2018). STEM tomography of high‐pressure frozen and freeze‐substituted cells: A comparison of image stacks obtained at 200 kV or 300 kV. Histochemistry and Cell Biology, 150, 545–556. 30229291 10.1007/s00418-018-1727-0

[23] Downing, K. H. (1992). Automatic focus correction for spot‐scan imaging of tilted specimens. Ultramicroscopy, 46, 199–206. 1481271 10.1016/0304-3991(92)90015-c

[24] Von Ardenne, M. (1938). Das Elektronen‐Rastermikroskop. Theoretische Grundlagen. Zeitschrift für Physik, 108, 338.

[25] McBride, E. , Rao, A. , Zhang, G. , Hoyne, J. , Calco, G. , Kuo, B. , He, Q. , Prince, A. , Pokrovskaya, I. , Storrie, B. , Sousa, A. , Aronova, M. , & Leapman, R. (2018). Comparison of 3D cellular imaging techniques based on scanned electron probes: Serial block face SEM vs. axial bright‐field STEM tomography. Journal of Structural Biology, 202(3), 216–228. 29408702 10.1016/j.jsb.2018.01.012 PMC8349566

[26] Sousa, A. A. , Azari, A. A. , Zhang, G. , & Leapman, R. D. (2011). Dual‐axis electron tomography of biological specimens: Extending the limits of specimen thickness with bright‐field STEM imaging. Journal of Structural Biology, 174(1), 107–114. 21055473 10.1016/j.jsb.2010.10.017 PMC3056916

[27] Villinger, C. , Gregorius, H. , Kranz, C. , Höhn, K. , Münzberg, C. , Wichert, G. , Mizaikoff, B. , Wanner, G. , & Walther, P. (2012). FIB/SEM tomography with TEM‐like resolution for 3D imaging of high‐pressure frozen cells. Histochemistry and Cell Biology, 138, 549–556. 22918510 10.1007/s00418-012-1020-6

[28] Yakushevska, A. , Lebbink, M. , Geerts, W. , Spek, L. , van Donselaar, E. , Jansen, K. , Humbel, B. , Post, J. , Verkleij, A. , & Koster, A. (2007). STEM tomography in cell biology. Journal of Structural Biology, 159(3), 381–391. 17600727 10.1016/j.jsb.2007.04.006

[29] Moor, H. , Bellin, G. , Sandri, C. , & Akert, K. (1980). The influence of high pressure freezing on mammalian nerve tissue. Cell and Tissue Research, 209, 201–216. 6994890 10.1007/BF00237626

[30] Moor, H. (1987). Theory and practice of high pressure freezing. In Steinbrecht, R. A. , & Zierold, K . (Eds.), Cryotechniques in biological electron microscopy (pp. 175–191, Chapter 8). Springer.

[31] Fernández‐Moran, H. (1959). Electron microscopy of retinal rods in relation to localization of rhodopsin. Science, 129, 1284.

[32] Simpson, W. L. (1941). An experimental analysis of the Altmann technic of freezing‐drying. The Anatomical Record, 80(2), 173–189.

[33] Abdellatif, M. E. A. , Sinzger, C. , & Walther, P. (2018). Investigating HCMV entry into host cells by STEM tomography. Journal of Structural Biology, 204(3), 406–419. 30352275 10.1016/j.jsb.2018.10.007

[34] Rachel, R. , Meyer, C. , Klingl, A. , Gürster, S. , Heimerl, T. , Wasserburger, N. , Burghardt, T. , Küper, U. , Bellack, A. , Schopf, S. , Wirth, R. , Huber, H. , & Wanner, G. (2010). Chapter 3 – Analysis of the ultrastructure of archaea by electron microscopy. Methods in Cell Biology, 96, 47–69. 20869518 10.1016/S0091-679X(10)96003-2

[35] Walther, P. , Schmid, E. , & Höhn, K. (2013). High‐pressure freezing for scanning transmission electron tomography analysis of cellular organelles. In Taatjes, D. J. , & Roth, J. (Eds.), Cell imaging techniques: Methods and protocols (pp. 525–535). Humana Press. 10.1007/978-1-62703-056-4_28 23027022

[36] Wilkes, M. , Madej, M. G. , Kreuter, L. , Rhinow, D. , Heinz, V. , De Sanctis, S. , Ruppel, S. , Richter, R. M. , Joos, F. , Grieben, M. , Pike, A. C. W. , Huiskonen, J. T. , Carpenter, E. P. , Kühlbrandt, W. , Witzgall, R. , & Ziegler, C. (2017). Molecular insights into lipid‐assisted Ca^2+^ regulation of the TRP channel Polycystin‐2. Nature Structural & Molecular Biology, 24(2), 123–132. 10.1038/nsmb.3357 28092368

[37] Buser, C. , & Walther, P. (2008). Freeze‐substitution: The addition of water to polar solvents enhances the retention of structure and acts at temperatures around –60°C. Journal of Microscopy, 230(2), 268–277. 18445157 10.1111/j.1365-2818.2008.01984.x

[38] Walther, P. , & Ziegler, A. (2002). Freeze substitution of high‐pressure frozen samples: The visibility of biological membranes is improved when the substitution medium contains water. Journal of Microscopy, 208(1), 3–10. 12366592 10.1046/j.1365-2818.2002.01064.x

[39] Luther, P. K. (2006). Sample shrinkage and radiation damage of plastic sections. In Frank, J. (Ed.), Electron tomography (2nd ed.). Springer Science+Business Media.

[40] Saxton, W. O. , Baumeister, W. , & Hahn, M. (1984). Three‐dimensional reconstruction of imperfect two‐dimensional crystals. Ultramicroscopy, 13, 57–70. 6382732 10.1016/0304-3991(84)90057-3

[41] Kremer, J. R. , Mastronarde, D. N. , & McIntosh, J. R. (1996). Computer visualization of three‐dimensional image data using IMOD. Journal of Structural Biology, 116(13), 71–76. 8742726 10.1006/jsbi.1996.0013

[42] McIntosh, R. , Nicastro, D. , & Mastronarde, D. (2005). New views of cells in 3D: An introduction to electron tomography. TRENDS in Cell Biology, 15(1), 43–51. 15653077 10.1016/j.tcb.2004.11.009

[43] Pintilie, G. D. , Zhang, J. , Goddard, T. D. , Chiu, W. , & Gossard, D. C. (2010). Quantitative analysis of cryo‐EM density map segmentation by watershed and scale‐space filtering, and fitting of structures by alignment to regions. Journal of Structural Biology, 170(3), 427–438. 20338243 10.1016/j.jsb.2010.03.007 PMC2874196

[44] Pettersen, E. F. , Goddard, T. D. , Huang, C. C. , Couch, G. S. , Greenblatt, D. M. , Meng, E. C. , & Ferrin, T. E. (2004). UCSF chimera – A visualization system for exploratory research and analysis. Journal of Computational Chemistry, 25, 1605–1612. 15264254 10.1002/jcc.20084

[45] Lum, P. Y. , & Wright, R. (1995). Degradation of HMG‐CoA reductase‐induced membranes in the fission yeast, *Schizosaccharomyces pombe* . Journal of Cell Biology, 131(1), 81–94. 7559789 10.1083/jcb.131.1.81 PMC2120600

[46] Borgese, N. , Francolini, M. , & Snapp, E. (2006). Endoplasmic reticulum architecture: Structures in flux. Current Opinion in Cell Biology, 18(4), 358–364. 16806883 10.1016/j.ceb.2006.06.008 PMC4264046

[47] Baumann, O. , & Walz, B. (2001). Endoplasmic reticulum of animal cells and its organization into structural and functional domains. In ( eon, K. W. Ed.), International review of cytology – A survey of cell biology (Vol. 205). Academic Press. 10.1016/s0074-7696(01)05004-5 11336391

[48] Xu, F. , Du, W. , Zou, Q. , Wang, Y. , Zhang, X. , Xing, X. , Li, Y. , Zhang, D. , Wang, H. , Zhang, W. , Hu, X. , Liu, X. , Liu, X. , Zhang, S. , Yu, J. , Fang, J. , Li, F. , Zhou, Y. , Yue, T. , & Yu, L. (2020). COPII mitigates ER stress by promoting formation of ER whorls. Cell Research, 31, 141–156. 32989223 10.1038/s41422-020-00416-2 PMC8026990

[49] Brill, A. L. , Fischer, T. T. , Walters, J. M. , Marlier, A. , Sewanan, L. R. , Wilson, P. C. , Johnson, E. K. , Moeckel, G. , Cantley, L. G. , Campbell, S. G. , Nerbonne, J. M. , Chung, H. J. , Robert, M. E. , & Ehrlich, B. E. (2020). Polycystin 2 is increased in disease to protect against stress‐induced cell death. Scientific Reports, 10(386), 1–15. 31941974 10.1038/s41598-019-57286-x PMC6962458

[50] Pathak, R. K. , Luskey, K. L. , & Anderson, R. G. W. (1986). Biogenesis of the crystalloid endoplasmic reticulum in UT‐1 cells: evidence that newly formed endoplasmic reticulum emerges from the nuclear envelope. Journal of Cell Biology, 102, 2158–2168. 3711144 10.1083/jcb.102.6.2158 PMC2114246

[51] Masaki, R. , Yamamoto, A. , & Tashiro, Y. (1994). Microsomal aldehyde dehydrogenase is localized to the endoplasmic reticulum via its carboxyl‐terminal 35 amino acids. Journal of Cell Biology, 126(6), 1407–1420. 8089174 10.1083/jcb.126.6.1407 PMC2290952

[52] Griffiths, G. , Warren, G. , Quinn, P. , Mathieu‐Costello, O. , & Hoppeler, H. (1984). Density of newly synthesized plasma membrane proteins in intracellular membranes. I . Stereological studies. Journal of Cell Biology, 98, 2133–2141. 6563037 10.1083/jcb.98.6.2133 PMC2113041

[53] Baumann, O. , & Walz, B. (1989). Topography of Ca^2+^‐sequestering endoplasmic reticulum in photoreceptors and pigmented glial cells in the compound eye of the honeybee drone. Cell and Tissue Research, 255, 511–522.

[54] Patergnani, S. , Suski, J. M. , Agnoletto, C. , Bononi, A. , Bonora, M. , De Marchi, E. , Giorgi, C. , Marchi, S. , Missiroli, S. , Poletti, F. , Rimessi, A. , Duszynski, J. , Wieckowski, M. R. , & Pinton, P. (2011). Calcium signaling around mitochondria associated membranes (MAMs). Cell Communication and Signaling, 9(19), 10.1186/1478-811X-9-19 PMC3198985 21939514

