# Supplementary material for: Application of STEM tomography to investigate smooth ER morphology under stress conditions
Source: J Microsc. 2025 Aug 12;299(3):228–41. doi: 10.1111/jmi.70020 (PMC12352019; doi:10.1111/jmi.70020)
Supplement: Supplementary file 1 — Supporting Information [file JMI-299-228-s001.docx]

**Supplementary figures:**


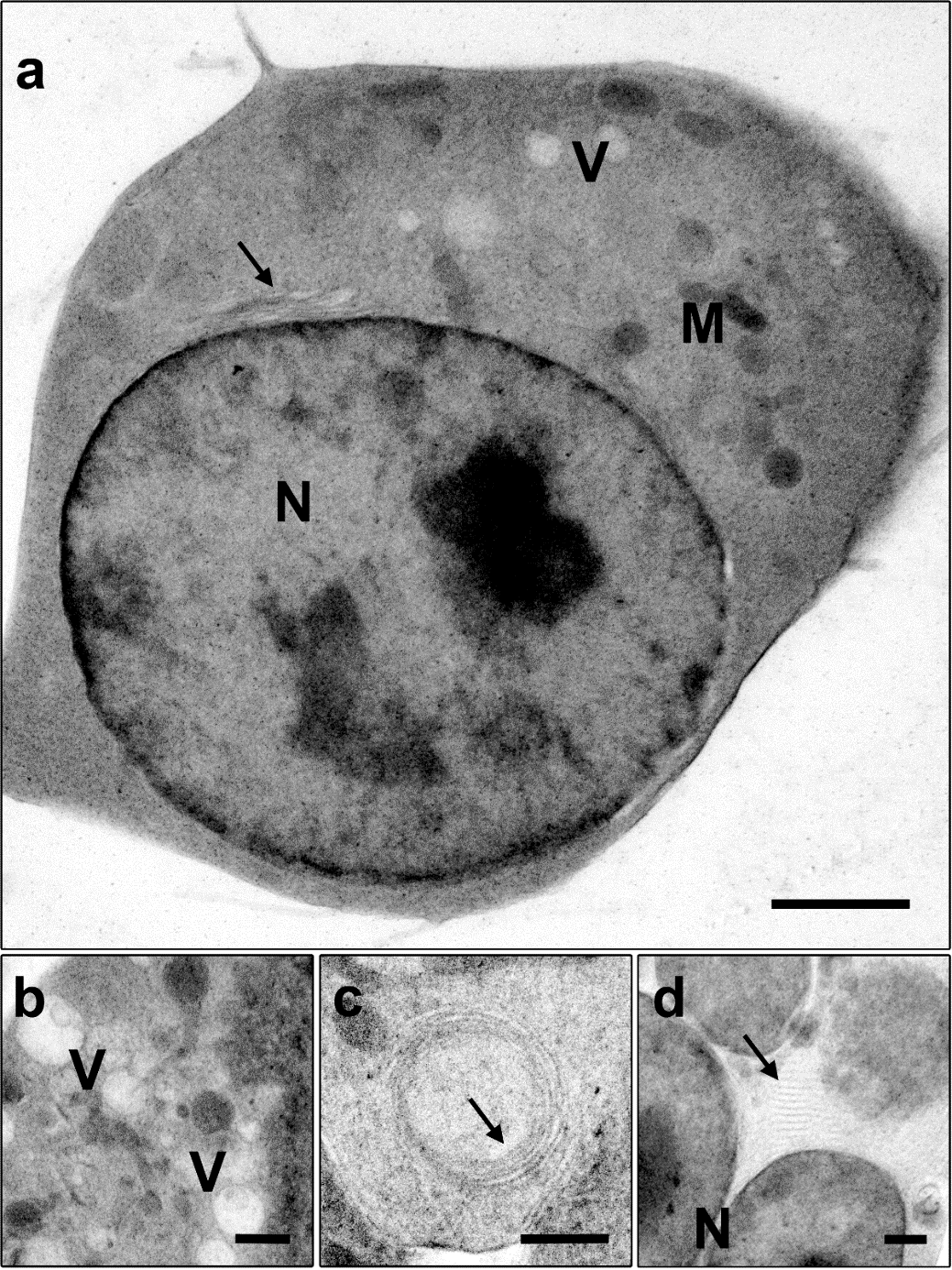


**Supplementary Figure 1:** Morphology of HEK cells upon over-expression of PC-2

**a)** TEM of an 800 nm section of a HEK293S GnTI^-^ cell after 72 h of overexpression of WT PC-2 proofs good ultra-structural sample preservation, evident from clearly confined electron-dense mitochondria (M), electron-transparent vesicles (V), membraneous structures (arrow) and the well-defined nucleus (N). Scale bar = 2000 nm. **b, c and d)** Zooms on different areas in the sample reveal the presence of diverse membraneous organelles, such as vesicles (b), putative lamellar (c) and tubular (d) OSER structures. Scale bars = 1000 nm.


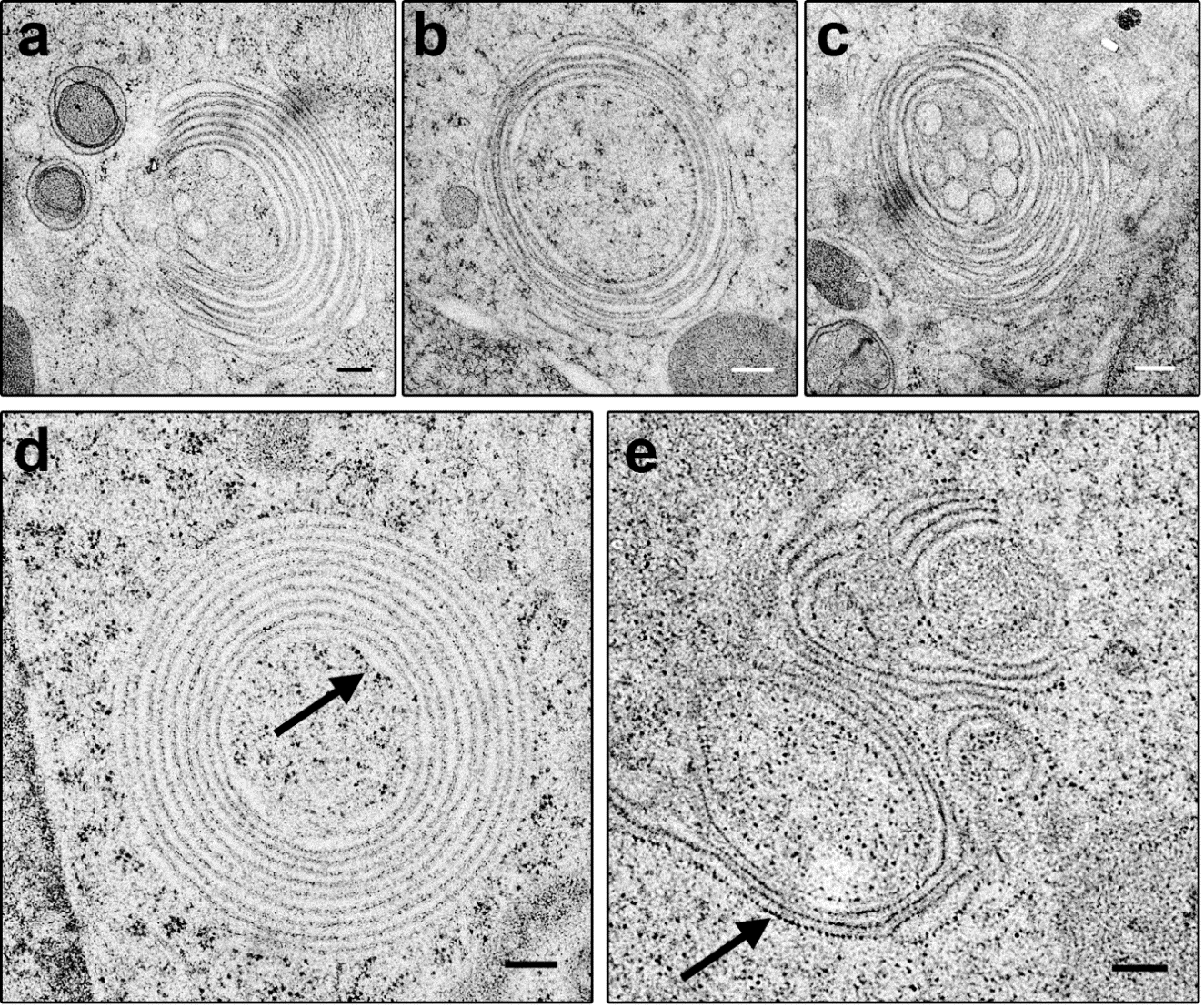


**Supplementary Figure 2:** ER whorl morphology

TEM of 50 nm sections of high-pressure frozen, freeze-substituted, OsO4 (a - c) or UAc / PbCi (d, e) stained and resin-embedded HEK293S GnTI- cells after 72 h of over-expression of WT PC-2. **a-c)** Examples of whorl structures. Whorls are often found in close association to vesicles and reach a diameter of up to 2 μm. The ER membranes exhibit regular spacing and enclose electron-dense cytoplasmic material. They occur as open (a) or closed (b, c) circles. **d, e)** In UAc stained and PbCi contrasted samples, electron dense spots (arrows) can be found lining up on some ER membranes, putatively representing ribosome-decorated rER. Scale bars = 200 nm.


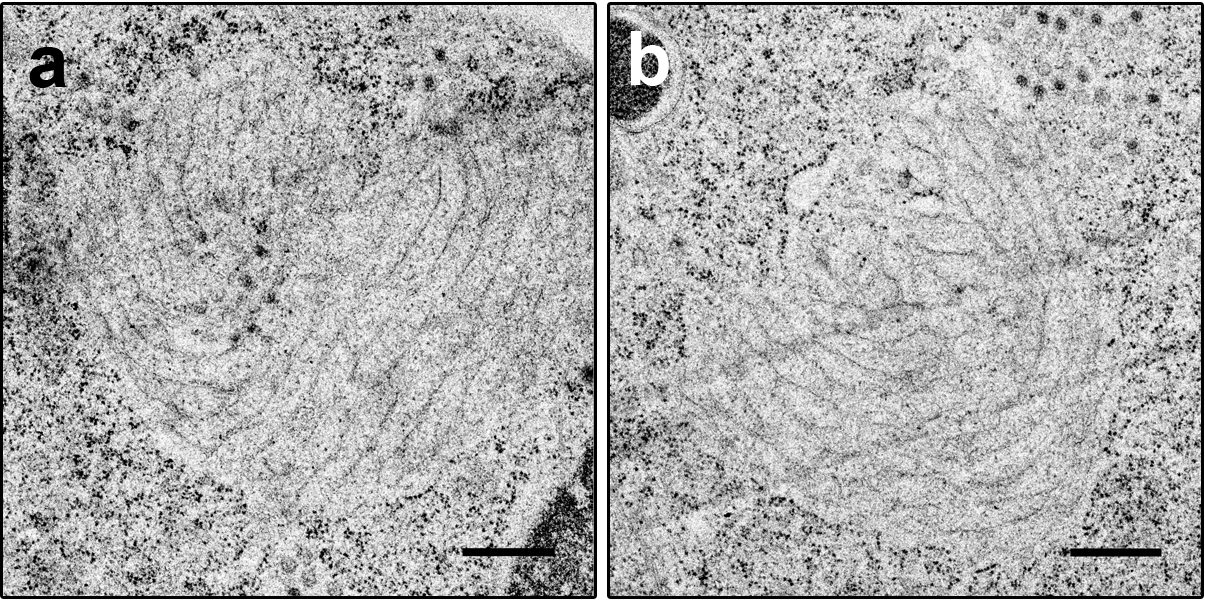


**Supplementary Figure 3:** Crystalloid-ER morphology

**a, b)** Morphology of putative crystalloid ER structures on 50 nm sections of high-pressure frozen, freeze-substituted, resin-embedded and UAc / PbCi stained HEK293S GnTI- cells after 72 h of overexpression of WT PC-2. The examples illustrate the difficulty of identifying the symmetric tubular arrays in non-perfect cross sections. Scale bars = 500 nm.


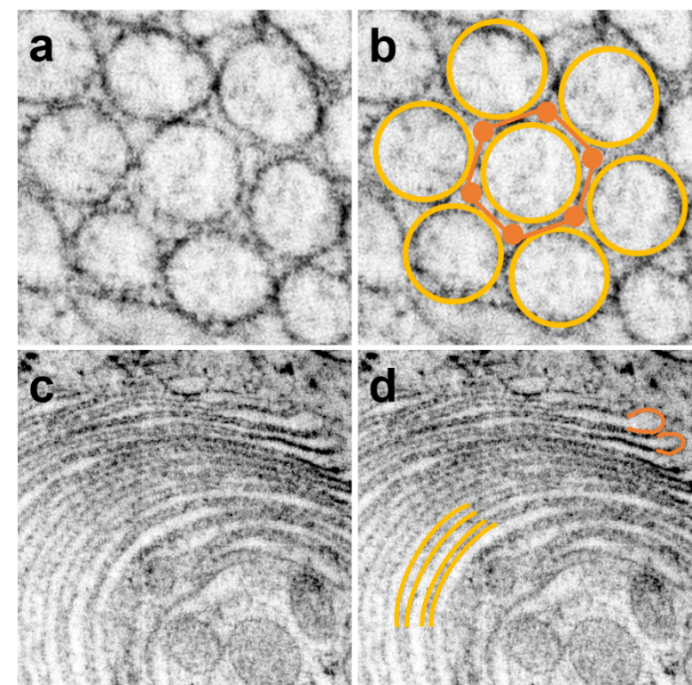


**Supplementary Figure 4:** Crystalloid-ER and whorl symmetry

**a)** Detail of a crystalloid-ER tube in cross-section, surrounded by six other tubes. The hexagonal-symmetric arrangement of the tubes is indicated in **b)** Tubes are depicted in gold, the tubes’ interspaces are indicated in orange. **c)** Detail of a cross-section in a whorl, highlighting the regular array of stacked membranes and the narrow terminal turns of the ER membranes. **d)** Stacked membranes feature a steady, but low curvature (gold), while the terminal membrane turns exhibit extreme inverse curvature (orange).


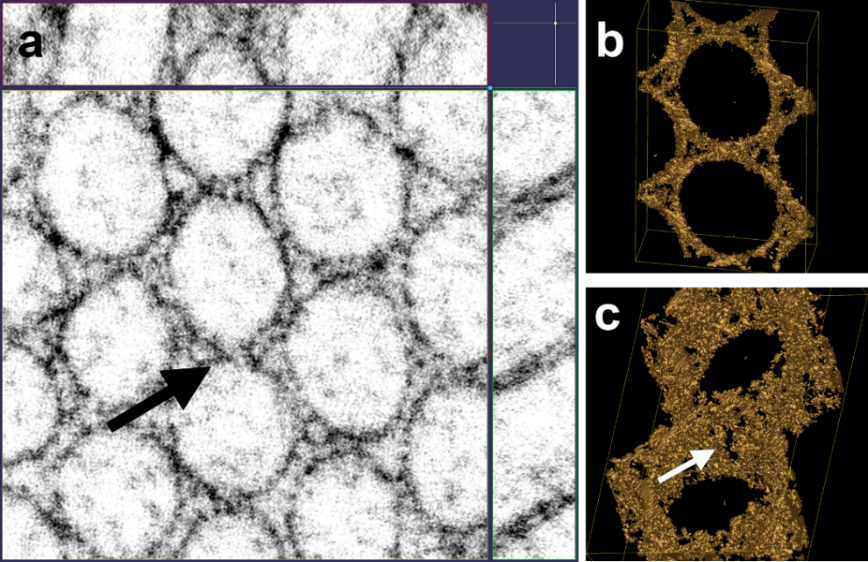


**Supplementary Figure 5:** Crystalloid-ER tube connection

**a)** Slice through a reconstructed crystalloid-ER tomogram in the X-Y-plane and the X-Z- (right) and Y-Z- (top) planes, respectively. A connection between two neighboring ER tubes is marked by an arrow. **b)** Isosurface depiction of the two connected tubes, illustrating the threshold-based tracing of the ER membranes. **c)** A tilted view of the same sub-volume, revealing a putative, narrow connection between the tubes (arrow).

**Supplementary tables:**

**Supplementary table 1:** Freeze substitution protocol

| **temperature** | **step** | **solution** | **incubation time(s)** |
| --- | --- | --- | --- |
| -140 °C | stepwise substitution of intracellular H_2_O, mild chemical fixation and en-bloc staining | substitution solution | 1 x 30 min |
| -140 °C to -90 °C |  | substitution solution | 1 x 3 h |
| -90 °C |  | substitution solution | 1 x 4 h |
| -90 °C to -60 °C |  | substitution solution | 1 x 3 h |
| -60 °C |  | substitution solution | 1 x 4 h |
| -60 °C to -30 °C |  | substitution solution | 1 x 3 h |
| -30 °C |  | substitution solution | 1 x 4 h |
| -30 °C to 0 °C |  | substitution solution | 1 x 3 h |
| 0 °C |  | substitution solution | 1 x 3 h |
| 0 °C | removal of substitution solution | Acetone p. a. | 2 x 10 min |
| 0 °C to 4 °C |  | Acetone p. a. | 1 x 20 min |
| 4 °C to 25 °C | removal of Acetone and stepwise infil­tration with Epon^TM^ 812 substitute em-bedding resin | Acetone / resin 2 + 1 | 1 x 1 h |
| 25 °C |  | Acetone / resin 2 + 1 | 1 x 1 h |
| 25 °C |  | Acetone / resin 1 + 1 | 1 x 2 h |
| 25 °C |  | Acetone / resin 1 + 2 | 1 x 16 h |
| 30 °C |  | resin (fresh) | 1 x 2 h |
| 60 °C | resin polymerization | resin | 1 x 2 d |

**Supplementary table 2:** Tilt scheme for dual-axis tomography

|  | **purpose** | **tasks** | **acquisition scheme** | |
| --- | --- | --- | --- | --- |
| series 1 | walking up | focusing  tracking | 0° to +66° | 5° increment |
| series 2 | data acquisition | focusing  tracking  exposure | +66° to +58° | 1.0° increment |
|  |  |  | +58° to +15° | continuously increasing increment |
|  |  |  | +15° to -15° | 2.0° increment |
|  |  |  | -15° to -58° | continuously decreasing increment |
|  |  |  | -58° to -66° | 1.0° increment |
| series 3 | walking down | focusing  tracking | -66° to 0° | 5° increment |
